# Prospective, multicenter study of pleural adhesion in repeated pulmonary surgery

**DOI:** 10.1186/1749-8090-10-S1-A343

**Published:** 2015-12-16

**Authors:** Yoshihiro Miyata, Hiroshi Date, Mitsugu Omasa, Kenji Suzuki, Kazuya Takamochi, Seiki Hasegawa, Nobuyuki Kondo, Morihito Okada

**Affiliations:** 1 Surgical Oncology, Hiroshima University, Hiroshima, Japan; 2 Kyoto University, Kyoto, Japan; 3 Juntendo University School of Medicine, Tokyo, Japan; 4 Hyogo College of Medicine, Mukogawa, Japan

## Background/Introduction

Pleural adhesion (PA) complicates repeated pulmonary surgery.

## Aims/Objectives

Herein, we prospectively investigated the degree of PA in the second thoracotomy to assess the impact of the previous ipsilateral thoracic procedure.

## Method

Seventy patients, with a median age of 67 years, undergoing a second thoracotomy with complete medical records of the previous thoracotomy from 4 institutions were included in this study. The mean interval from the first operation to the second operation was 20 months (1-105 months). The site and the extent of adhesion as well as duration and amount of bleeding while dissecting the adhesion were recorded.

## Results

Fifty-four (76%) patients had PA to the chest wall and 10 (14%) had dense and total PA in the thoracic cavity. Patients with PA experienced more bleeding (215 vs. 29.3 g), a longer drainage period (4.7 vs. 2.3 days), and higher post-operative morbidity (28% vs. 6%) than those without PA in the second operation. According to ROC analysis, cut-off values for operation duration, amount of bleeding, and length of thoracotomy incision in the initial operation for predicting PA were 75 minutes, 10 g, and 6.0 cm, respectively. On the basis of the univariate analysis for PA, operation time >75 minutes, bleeding >10 g, thoracotomy incision length >6.0 cm, and segmentectomy or lobectomy in the initial surgery were significantly associated with PA. Multivariate analysis revealed only thoracotomy incision length >6.0 cm was the independent predictor for PA (p = 0.0065). All patients with thoracotomy incision length >6.0 cm showed PA, but 54% of patients with thoracotomy incision length <6.0 cm. On the other hand, thoracotomy incision length was not associated with dense and total PA. Multivariate analysis identified only post-operative drainage period >5 days in the initial surgery to be the independent predictor for dense and total PA in the thoracic cavity (p = 0.016).

## Discussion/Conclusion

Long thoracotomy incision length in the initial surgery is a predictor for PA at the second surgery. Dense and total PA might be caused by post-operative inflammation in the thoracic cavity due to prolonged air leakage.

